# Thermodynamic properties of 5(nitrophenyl) furan-2-carbaldehyde isomers

**DOI:** 10.1186/s13065-015-0144-x

**Published:** 2015-12-09

**Authors:** Volodymyr Dibrivnyi, Iryna Sobechko, Marian Puniak, Yuriy Horak, Mykola Obushak, Yuriy Van-Chin-Syan, Marshalek Andriy, Nadiia Velychkivska

**Affiliations:** National University “LvivPolytechnic”, S. Bandery Str.,12, Lviv, 79013 Ukraine; Ivan Franko National University of Lviv, Kyryla and Mefodiya Str., 6, Lviv, 79005 Ukraine; Charles University in Prague, Ovocný trh 3-5, Prague 1, 116 36 Czech Republic; P. Kobylytsi str. 19/40, Lviv, 79053 Ukraine

**Keywords:** 5(2-Nitrophenyl)-furan-2-carbaldehydes, Vapor pressure, Combustion enthalpy, Formation enthalpy, Vaporization enthalpy, Sublimation enthalpy, Isomerisation enthalpy

## Abstract

**Background:**

The aim of the current work was to determine thermo dynamical properties of 5(2-nitro phenyl)-furan-2-carbaldehyde, 5(3-nitro phenyl)-furan-2-carbaldehyde and 5(4-nitro phenyl)-furan-2-carbaldehyde.

**Results:**

The temperature dependence of saturated vapor pressure of 5(2-nitro phenyl)-furan-2-carbaldehyde, 5(3-nitro phenyl)-furan-2-carbaldehyde and 5(4-nitro phenyl)-furan-2-carbaldehyde was determined by Knudsen’s effusion method. The results are presented by the Clapeyron–Clausius equation in linear form, and via this form, the standard enthalpies, entropies and Gibbs energies of sublimation and evaporation of compounds were calculated at 298.15 K. The standard molar formation enthalpies of compounds in crystalline state at 298.15 K were determined indirectly by the corresponding standard molar combustion enthalpy, obtained using bomb calorimetry combustion.

**Conclusions:**

Determination of the thermodynamic properties for these compounds may contribute to solving practical problems pertaining optimization processes of their synthesis, purification and application and it will also provide a more thorough insight regarding the theoretical knowledge of their nature.

**Graphical abstract:**
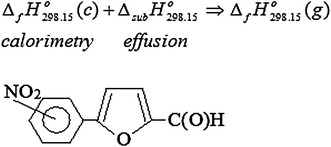
Generalized structural formula of investigated compounds and their formation enthalpy determination scheme in the gaseous state

## Background

Arylfurane compounds (such as aldehydes) exhibit different biological activity (antimicrobial, anticonvulsant, anticancer, tuberculostatic, etc.), due to which they are used as medications [1–4]. Obviously, the presence of nitro and carbonyl groups in the investigated compounds extends possibilities of their practical usage. The reaction of 5-aryl-2-furane carbaldehydes in three-component cyclization with urea or thiourea and furoilacetic ester in the Bidginelli [5] reaction was already studied. This multicomponent reaction is very promising because it saves resources and promotes the concept of “green” chemistry. The search of promising reactions involving 5-aryl-2-furane carbaldehydes and the need of a deeper understanding of their biochemical functions require their thermodynamic properties to be studied.

The evaporation and sublimation enthalpies, entropy and Gibbs energy, determined in the present work, contain information about the energy of intermolecular interactions in the investigated compounds. The formation enthalpy of a substance in the gaseous state, calculated on the basis of the vaporization enthalpy and the formation enthalpy of in condensed form, contains information about the internal interactions between atoms in the molecule. Joint analysis of these properties for a number of compounds will reveal many theoretically important patterns of mutual influence of atoms in a molecule and enable to calculate the enthalpy of formation of free radicals, energy relations, tension, cyclization, determine the additive group contributions to the scheme.

Thermodynamic properties allow finding the most energetically favorable ways of synthesis of compounds with the maximum economic benefit. Temperature dependence of vapor pressure has its own practical value for calculating the parameters of individual stages of the synthesis.

## Results and discussion

### Effusion measurements

**Fig. 1:**
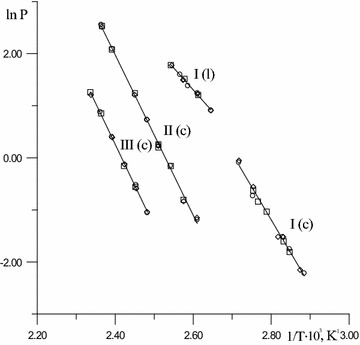
Clausius–Clapeyron equation for isomeric aldehydes in liquid (l) and crystalline (c) state in form lnP = f(1/T). *Circle* sample A; *square* sample B; *diamond* sample C

Primary effusion measurement results, including the saturated vapor pressure P of researched compounds are shown in Table 1. The vapor pressures of substance (I) in the liquid and solid state and for substances (II–III) in the solid state were measured. Coincidence of the vapor pressures of aldehydes samples obtained after recrystallization with different multiplicities (A, B, C), (Fig. 1) accurately indicates that our installation is suitable for thermodynamic measurements.

**Table 1:** Results of effusion measurements of investigated substances

Sample	*T*, К	*m* × 10^3^, g	τ × 10^−3^, s	*P*_k_, Pa	*P*, Pa	Sample	*T*, К	*m* × 10^3^, g	τ × 10^−3^, s	*P*_k_, Pa	*P*, Pa
5-(2-nitrophenyl)-furan-2-carbaldehyde, l. T_fus_ = 368.3 ± 1.0 К						5-(2-nitrophenyl)-furan-2-carbaldehyde, cr. T_fus_ = 368.3 ± 1.0 К					
A	378.0	6.60	3.62	2.18	2.48	A	346.6	0.91	10.82	0.095	0.108
	383.0	9.14	3.63	3.03	3.46		351.2	0.95	7.22	0.152	0.173
	386.6	5.26	1.82	3.49	3.98		353.4	0.90	5.42	0.192	0.218
	389.7	6.51	1.82	4.34	4.95		363.2	1.32	3.62	0.421	0.480
B	382.7	4.45	1.83	2.93	3.34		368.0	2.46	3.62	0.801	0.912
	387.9	6.01	1.81	4.00	4.56	B	347.9	0.80	9.02	0.102	0.116
	393.3	7.76	1.82	5.20	5.93		353.2	0.61	3.62	0.191	0.218
C	378.0	6.60	3.62	2.18	2.48		354.9	0.89	5.42	0.192	0.219
	383.0	9.01	3.62	2.99	3.41		363.1	1.45	3.63	0.468	0.533
	388.5	5.92	1.83	3.92	4.46		368.0	2.56	3.62	0.830	0.947
	393.0	7.74	1.82	5.19	5.91	C	351.2	0.90	7.21	0.144	0.164
5-(3-nitrophenyl)-furan-2-carbaldehyde, cr., T_fus_ = 428.6 ± 1.0 К							353.1	1.11	7.21	0.176	0.201
A	383.1	1.20	5.42	0.266	0.303		358.6	1.44	5.41	0.312	0.355
	393.4	2.23	3.61	0.750	0.855		361.5	1.75	5.41	0.378	0.431
	398.3	3.25	3.62	1.10	1.26		363.1	1.54	3.62	0.501	0.572
	403.0	5.35	3.62	1.82	2.08	5-(4-nitrophenyl)-furan-2-carbaldehyde, cr., T_fus_ = 479.8 ± 0.8 К					
	418.1	15.5	2.73	7.11	8.11	A	402.8	1.34	5.42	0.305	0.347
	423.0	24.3	2.72	11.3	12.9		407.6	2.27	5.42	0.520	0.592
B	388.3	1.75	5.41	0.391	0.446		418.2	3.77	3.62	1.31	1.49
	393.3	2.22	3.62	0.748	0.853		423.5	6.05	3.61	2.11	2.41
	398.3	3.15	3.61	1.07	1.22	B	407.9	2.19	5.41	0.502	0.572
	398.3	3.34	3.62	1.13	1.29		412.8	2.18	3.61	0.751	0.856
	408.1	8.85	3.62	3.04	3.46		423.1	5.90	3.62	2.06	2.35
	418.2	15.2	2.71	7.03	8.02		428.1	8.78	3.61	3.09	3.52
	422.7	23.8	2.72	11.0	12.6	C	403.0	1.36	5.41	0.310	0.353
C	383.2	1.25	5.42	0.278	0.317		407.5	2.13	5.41	0.487	0.555
	388.3	1.70	5.42	0.380	0.433		412.6	2.25	3.62	0.774	0.882
	398.3	3.31	3.62	1.12	1.28		418.0	3.74	3.62	1.30	1.48
	403.0	5.34	3.62	1.82	2.07		427.8	8.36	3.62	2.93	3.34
	408.2	8.55	3.62	2.93	3.34						
	422.8	23.4	2.72	10.9	12.4

The measurement results were processed by the method of least squares and presented as a linear equation: ln P (Pa) = A + B/T with correlation coefficient *ρ*, by means of which the standard molar enthalpies $\varDelta_{cr,l}^{g} H_{m}^{o} (\left\langle T \right\rangle )$ = *B·R* and standard molar entropies $\varDelta_{cr,l}^{g} S_{m}^{o} (\left\langle T \right\rangle )$ = *A·R* − *R·lnP°* (Pº = 0.1 MPa) of sublimation (vaporization) were calculated at average temperatures of measurement interval $\left\langle T \right\rangle$ (Table 2).

**Table 2:** Coefficients of a linear equation: ln P (Pa) = A + B/T, standard vaporization and sublimation enthalpies and entropies of investigated substances

Substance	*T*_m,_ К	*A*	−*B*, 10^−2^, К	*ρ,* %	$\varDelta_{cr,l}^{g} H_{m}^{o} (\left\langle T \right\rangle )$, kJ mol^−1^	$\varDelta_{cr,l}^{g} S_{m}^{o} (\left\langle T \right\rangle )$, J mol^−1^ K^−1^
I (cr → g)	357.3	34.3 ± 1.9	126.6 ± 6.9	0.9911	105.2 ± 5.7	189 ± 16
I (l → g)	385.7	23.2 ± 1.0	84.2 ± 4.0	0.9952	70.0 ± 3.3	97.1 ± 8.3
II (cr → g)	403.0	38.91 ± 0.76	153.8 ± 3.5	0.9981	127.9 ± 2.9	227.7 ± 6.3
III (cr → g)	415.5	38.19 ± 0.69	158.1 ± 2.9	0.9988	131.4 ± 2.4	221.7 ± 5.7

Standard enthalpies and entropies of sublimation and vaporization can be adjusted to 298.15 K by the equations:

1 $$\varDelta_{cr,l}^{g} H_{m}^{o} (298.15{\text{K}}) = \varDelta_{cr,l}^{g} H_{m}^{o} (\left\langle T \right\rangle ) + \varDelta_{cr,l}^{g} Cp_{m}^{o} (298.15K) \cdot (\left\langle T \right\rangle - 298.15)$$

2 $$\varDelta_{cr,l}^{g} S_{m}^{o} (298.15{\text{K}}) = \varDelta_{cr,l}^{g} S_{m}^{o} (\left\langle T \right\rangle ) + \varDelta_{cr,l}^{g} Cp_{m}^{o} (298.15K) \cdot \ln (\left\langle T \right\rangle /298.15)$$

The changes of standard phase transitions heat capacity values $\varDelta_{cr,l}^{g} Cp_{m}^{o}$ at indicated temperature ranges for the probed compounds are unknown. Therefore, the Eqs. (3–6) were used to calculate the enthalpies and entropies of sublimation and vaporization at 298.15 K [6].

3 $$\varDelta_{cr}^{g} H_{m}^{o} (298.15K)/({\text{kJ mol}}^{ - 1} ) = \varDelta_{cr}^{g} H_{m}^{o} (\left\langle T \right\rangle ) + [0.75 + 0.15Cp_{{m_{cr} }}^{o} (298.15K)] \cdot (\left\langle T \right\rangle - 298.15)$$

4 $$\varDelta_{l}^{g} H_{m}^{o} (298.15{\text{K}})/({\text{kJ mol}}^{ - 1} ) = \varDelta_{l}^{g} H_{m}^{o} (\left\langle T \right\rangle ) + [10.58 + 0.26Cp_{{m_{l} }}^{o} (298.15{\text{K}})] \cdot (\left\langle T \right\rangle - 298.15)$$

5 $$\begin{aligned} \varDelta_{cr}^{g} S_{m}^{o} (298.15{\text{K}})/({\text{J }}({\text{mol K}})^{ - 1} ) \hfill \\ = \varDelta_{cr}^{g} S_{m}^{o} (\left\langle T \right\rangle ) + [0.75 + 0.15Cp_{{m_{cr} }}^{o} (298.15{\text{K}})] \cdot \ln (\left\langle T \right\rangle /298.15) \hfill \\ \end{aligned}$$

6 $$\begin{aligned} \varDelta_{l}^{g} S_{m}^{o} (298.15{\text{K}})/({\text{J }}({\text{mol K}})^{ - 1} ) \hfill \\ = \varDelta_{l}^{g} S_{m}^{o} (\left\langle T \right\rangle ) + [10.58 + 0.26Cp_{{m_{l} }}^{o} (298.15{\text{K}})] \cdot \ln (\left\langle T \right\rangle /298.15) \hfill \\ \end{aligned}$$

The standard heat capacity changes during sublimation and vaporization, represented by empirical factors in square brackets Eqs. (3–6), were obtained by the authors [7] using statistical analysis of a large set of experimental values. Heat capacities in solid $Cp_{{m_{cr} }}^{o}$ and liquid $Cp_{{m_{l} }}^{o}$ states were calculated by the additive method [6] and were (J mol^−1^): $Cp_{{m_{cr} }}^{o}$(298.15 K) = 289.3; $Cp_{{m_{l} }}^{o}$(298.15) = 332.9.

Standard Gibbs energies of sublimation and vaporization at 298.15 K were calculated by the equation:

7 $$\varDelta_{cr,l}^{g} G_{m}^{o} (298.15{\text{K}}) = \varDelta_{cr,l}^{g} H_{m}^{o} (298.15{\text{K}}) - 298.15 \cdot \varDelta_{cr,l}^{g} S_{m}^{o} (298.15{\text{K}})$$

Standard sublimation and vaporization enthalpies, entropies and Gibbs energies at 298.15 K are shown in Table 3.

**Table 3:** Standard enthalpies, entropies and Gibbs energies of sublimation and vaporization of 5(nitrophenyl)-2-carbaldehydes at 298.15 K

Substance	$\varDelta_{cr,l}^{g} H_{m}^{o} (298.15{\text{K}})$ kJ mol^−1^	$\varDelta_{cr,l}^{g} S_{m}^{o} (298.15{\text{K}})$, J mol^−1^ K^−1^	$\varDelta_{cr,l}^{g} G_{m}^{o} (298.15{\text{K}})$, kJ mol^−1^
I (cr → g)	107.8 ± 6.7	197 ± 25	49.1 ± 6.9
I (l → g)	78.5 ± 4.3	121 ± 18	42.0 ± 4.5
II (cr → g)	132.5 ± 3.9	241 ± 16	60.7 ± 4.1
III (cr → g)	135.9 ± 3.5	236 ± 16	65.4 ± 3.7

Prior to this, the reliability of the effusion installation was checked by etalon benzoic acid brand K-1 by a series of forty experiments for benzoic acid. The dependence of saturated vapor pressure on temperature has the form: ln P = (34.84 ± 0.16) − (10,882 ± 55) × 1/T; the correlation coefficient ρ = 0.994. The average value of the standard enthalpy of sublimation in the temperature range of (322.7–353.6) K was $\varDelta_{cr}^{g} H_{m}^{o} (T_{m} )$ = 90.48 ± 0.46 kJ mol^−1^. In order to adjust the standard enthalpy of sublimation to 298 K, using Eq. (1), standard heat capacity of benzoic acid at 298.15 J mol^−1^ K^−1^) in the solid $Cp_{{m_{cr} }}^{o}$ = 146.76 ± 0.32 [8] and gaseous $Cp_{{m_{g} }}^{o}$ = 103.47 [9] states were utilized. Good coincidence of benzoic acid’s sublimation enthalpy adjusted to 298.15 K $\varDelta_{cr}^{g} H_{m}^{o} (298.15)$ kJ/mol according to the Eq. (1) 91.39 ± 0.56, and Eq. (3) 92.21, with the recommended values 89.7 ± 1.0 [8], and 89.0 ± 4.0 [9] shows the absence of significant systematic errors in the effusion installation.

### Calorimetric measurements

Combustion of the investigated 5-(2-, 3- and 4- nitro phenyl)-furan-2-carbaldehydes is represented by reaction:

$${\text{C}}_{ 1 1} {\text{H}}_{ 7} {\text{O}}_{ 4} {\text{N}}_{{({\text{s}})}} + 10. 7 5 {\text{ O}}_{{ 2({\text{g}})}} = 1 1 {\text{ CO}}_{{ 2({\text{g}})}} + 3. 5 {\text{ H}}_{ 2} {\text{O}}_{{({\text{l}})}} + 0. 5 {\text{ N}}_{{ 2({\text{g}})}}$$

Combustion energy *∆*_*c*_*U*(*cpd*) of the investigated substances was calculated by the equation:

8 $$- \varDelta_{c} U(cpd) = (\varDelta U_{\varSigma } - \varDelta U_{fuse} - \varDelta U_{ter} - \varDelta U_{{HNO_{3} }} + \varDelta U_{carbon} )/m(cpd)$$

where *m(cpd)—*compound weight determined using gas analysis; *ΔU*_*Σ*_ = *ε(calor)*.∙*∆T*—total heat released in the experiment, *ε(calor)*—energy equivalent of the calorimetric system, *∆T*—real increase in temperature. Calculations were performed taking into account corrections for the combustion of cotton thread *ΔU*_*fuse*_, terylene container *ΔU*_*ter*_, soot to carbon dioxide *ΔU*_*carbon*_ and also for formation in a bomb nitric acid solution *ΔU*_*HNO3*_, using the following data for heats of combustion (J g^−1^): terylene 22,944.2 [10]; cotton thread 16,704.2 [10]; soot to carbon dioxide −32,763 [11]; formation of nitric acid 59 kJ mol^−1^ [11].

The results of determination of the aldehydes combustion energies *∆*_*c*_*U(cpd*) are listed in Table 4, which besides the above notation, m^exp^/m^cal^, also specifies combustion completion by carbon dioxide, that was obtained experimentally.

**Table 4:** Results of the experimental determination of combustion energies of the investigated compounds at 298.15 K

Sample	*m(cpd)*,g	*ΔT,* V	*ΔU*_*Σ*_, J	*ΔU*_*fuse*_, J	*ΔU*_*ter*_, J	*ΔU*_*HNO3*_, J	*ΔU*_*carbon*_, J	–*∆*_*c*_ *U(cpd*), J g^−1^	*m*_*exp*_ */m*_*calc*_
5(2-nitrophenyl)furan-2-carbaldehyde; −*∆*_*c*_ *U(cpd*)_*average*_ **=** 23,674 ± 31 J g^−1^									
A	0.32424	0.5592	8542.9	100.4	795.4	8.3	17.6	23,701	0.9964
A	0.30861	0.5368	8200.7	87.8	834.7	9.4	23.3	23,639	0.9996
A	0.35576	0.6065	9265.5	94.1	800.2	10.6	40.8	23,683	0.9972
B	0.27531	0.4826	7372.7	110.4	802.6	11.8	49.6	23,677	0.9968
C	0.30758	0.5366	8197.6	108.1	834.0	11.8	21.5	23,635	0.9994
C	0.35026	0.5968	9117.3	112.3	698.5	11.8	5.0	23,710	0.9994
5-(3-nitro phenyl)-furan-2-carbaldehyde; −*∆*_*c*_ *U(cpd*)_*average*_ **=** 23,633 ± 37 J g^−1^									
A	0.40261	0.6779	10,356.3	85.2	932.0	27.7	18.4	23,595	0.9901
A	0.37500	0.6246	9542.0	94.5	899.8	17.7	29.5	23,645	0.9953
B	0.29162	0.4984	7614.1	84.2	791.6	23.6	17.2	23,671	0.9952
B	0.32049	0.5496	8396.2	89.6	842.5	17.7	12.8	23,581	0.9987
C	0.35108	0.6020	9196.8	91.4	831.5	25.4	10.2	23,665	0.9940
C	0.45773	0.7687	11,743.4	87.3	969.5	17.9	22.4	23,640	0.9922
5-(4-nitro phenyl)-furan-2-carbaldehyde; −*∆*_*c*_ *U(cpd*)_*average*_ **=** 23,533 ± 31 J g^−1^									
A	0.34298	0.5852	8940.1	74.3	818.3	12.9	16.9	23,507	0.9986
B	0.35493	0.6003	9170.8	87.0	734.4	20.1	22.3	23,553	0.9990
B	0.39564	0.6757	10,322.7	101.3	946.3	14.8	30.5	23,520	0.9984
B	0.39158	0.6564	10,027.8	97.9	771.6	14.8	26.4	23,586	0.9929
C	0.37234	0.6233	9522.2	91.6	721.7	14.8	44.8	23,510	0.9983
C	0.41218	0.6907	10,551.8	100.5	782.4	14.8	31.3	23,522	0.9990

Coincidence within the accuracy of the installation of the combustion energies and completion of the aldehyde samples obtained after recrystallization of different multiplicities (A, B, C), (Table 4) is indicating their sufficient purity for use in the calorimeter. High rate of consistency of carbon dioxide content in substances (0.9901–0.9996) calculated by the formula, (the results of its experimental determination are shown by the Rossini method) can also serve as an indirect confirmation of the sufficient purity of the compounds.

The absence of significant systematic errors while measuring at the calorimetry installation was confirmed by the coincidence of our results of combustion enthalpy (kJ mol^−1^) of stilbene, purified by zone melting—7355.0 ± 4.0 [12], with recommended ones: −7358.8 [13]; −7357.1 ± 0.8 [14].

The standard combustion enthalpies $\varDelta {}_{c}H_{m}^{o} (cr)$(298.15 K) of aldehydes were calculated taking into account the correction for the volume expansion work ***∆****nRT* and the Washburn correction. Both corrections were calculated according to [11].The calculation of enthalpy formations in condensed phase was based on the following key values of $\varDelta {}_{f}H_{m}^{o} (298.15K)$, kJ mol^−1^: (−285.830 ± 0.042) (H_2_O, l.), (−393.514 ± 0.046) (CO_2_, g) [10];

Standard combustion $- \varDelta {}_{c}H_{m}^{o} (cr)$ and formation $- \varDelta {}_{f}H_{m}^{o} (cr)$ enthalpies of investigated aldehydes are listed in Table 5.

**Table 5:** Combustion and formation enthalpies of 5-(nitro phenyl)-furan-2-carbaldehydes in (kJ mol^−1^), at T = 298.15 K

Substance	$- \varDelta {}_{c}H_{m}^{o} (cr)$	$- \varDelta {}_{f}H_{m}^{o} (cr)$	$\varDelta_{cr}^{g} H_{m}^{o}$	$- \varDelta {}_{f}H_{m}^{o} (g)$
I	5137.0 ± 6.8	193.9 ± 6.8	107.8 ± 6.7	86.1 ± 9.7
II	5127.4 ± 8.0	203.5 ± 8.0	132.5 ± 3.9	71.0 ± 8.9
III	5106.4 ± 6.7	224.5 ± 6.7	135.9 ± 3.5	88.6 ± 7.6

## Experimental

### Materials

**Fig. 2:**
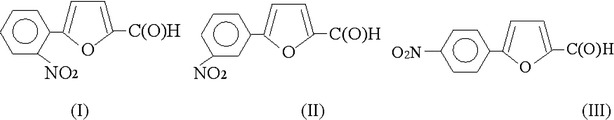
Molecular structures of 5-(2-nitro phenyl)-furan-2-carbaldehyde (**I**), 5-(3-nitrophenyl)-furan-2-carbaldehyde (**II**) and 5-(4-nitro phenyl)-furan-2-carbaldehyde (**III**)

Investigated 5-(2-nitrophenyl)-furan-2-carbaldehyde (I), 5-(3-nitrophenyl)-furan-2-carbaldehyde (II) and 5-(4-nitrophenyl)-furan-2-carbaldehyde (III) isomers are crystalline substances under normal conditions (Fig. 2).

The investigated compounds were synthesized via the reaction of furfural with arenediazonium chloride solution obtained by diazotization of the corresponding amine. Products were filtered and purified by double recrystallization from ethanol-dimethyl formamide mixture. Samples (A), (B) and (C) were used and were obtained after third, fourth and fifth sequential re-crystallization respectively. The identification of substances was confirmed by elemental analysis for carbon, hydrogen and nitrogen as well as by data of NMR spectroscopy. NMR^1^H spectra were recorded by using Varian 600 (600 MHz) device. Solvent—DMSO-d6. Chemical shifts (δ. pph) were determined in regards to the signal of DMSO (2.50 pph). Spectra data for the investigated substances are shown below:

**(I)**^1^H NMR (600 MHz. DMSO) δ 7.24 (d. *J* = 3.7 Hz. 1H. fur). 7.72 (d. *J* = 3.7 Hz. 1H. fur). 7.79 (t. *J* = 7.8. 1H. C_6_H_4_). 7.88 (t. *J* = 7.8 Hz. 1H. C_6_H_4_). 7.99 (d. *J* = 7.8. 1H. C_6_H_4_). 8.07 (d. *J* = 7.8 Hz. 1H. C_6_H_4_). 9.66 (s. 1H. CHO).

**(II)**^1^H NMR (600 MHz. DMSO) δ 7.62 (d. *J* = 3.7 Hz. 1H. fur). 7.75 (d. *J* = 3.7 Hz. 1H. fur). 7.85 (t. *J* = 8.0 Hz. 1H. C_6_H_4_). 8.31 (d. *J* = 8.3 Hz. 1H. C_6_H_4_). 8.35 (d. *J* = 8.4 Hz. 1H. C_6_H_4_). 8.65 (s. *J* = 1.8 Hz. 1H. C_6_H_4_). 9.72 (s. 1H. CHO).

**(III)**^1^HNMR (600 MHz. DMSO) δ 7.63 (d. *J* = 3.7 Hz. 1H. fur). 7.76 (d. *J* = 3.7 Hz. 1H. fur). 8.17 (d. *J* = 8.8 Hz. 2H. C_6_H_4_). 8.39 (d. *J* = 8.8 Hz. 2H.C_6_H_4_). 9.73 (s. 1H. CHO).

The purity of the investigated compounds was also controlled by TLC.

### Effusion measurements

Taking into account the low volatility of the analyzed substances, the temperature dependence of the saturated vapor pressure was determined by the integral Knudsen effusion method. The design of the apparatus has been adopted from [15]. Construction of the chamber, membrane and experimental procedure were conducted using the recommendations [16]. Effusional installation camera is the cylindrical cup made out of stainless steel. The material used in the chamber, is characterized by high thermal conductivity, but does not react with the samples in the condensed or vapor state. The camera is sealed using Teflon gaskets, nickel membrane, washer and nut. Copper washer provides reliable thermal contact with a nut and membrane. Hyperbolic shape of the washer top allows avoiding hypothermia of the membrane surface. This method does not require large mechanical efforts for sealing and does not deform membrane, moreover it is quite simple and reliable in operating within the temperature range of 278–398 K.

The membrane is made of nickel foil with the thickness of 0.05 mm. Effusional holes were obtained by drilling of the foil sandwiched between plates of transformer steel, so that the thickness of the hole edges after drilling membranes remained unchanged. After drilling holes were treated with nylon fishing line. Hole diameters were measured by an electron microscope REM 106I with accuracy of 0.0001 mm. Membrane with diameter d = 0.5903 mm was used for effusional research presented in this paper.

The crystalline sample of the substance (0.2–0.3) g was placed into the efusional camera and compressed by the steel punch to maximize thermal contact. After the cell was sealed, it was inserted in a copper block, where the air was pumped out by the system of forvacuum and paraoil pumps. Then the camera was filled with helium till the pressure of 0.1 MPa. The copper block was placed in the heated thermostat and heated to the necessary temperature maintained within ±0.5 K. After the sample was thermostated for 40 min, the chamber was vacuumed again. The moment when the residual pressure in the system was equal to 0.1 Pa (for 41 ± 10 s) was accepted as the beginning of experiment.

Effective time (estimated time of effusion in the steady state, in which the weight loss of the effunded substance is equal to that in the transient regime) was determined in separate experiments with benzoic acid and equals to 30 ± 5 s.

The moment of effusion chamber isolation from the vacuum system and its filling with helium under the pressure of 0.1 MPa was considered as the end of the experiment. After the camera had been pulled out and cooled to room temperature, it wasn’t weighted for additional 20 min for the purpose of air desorption. The weight of the efunded substance *m* was determined using analytical scales VLR-20 (±5 × 10^−6^ g) as the difference of the efusion camera weight before and after the experiment.

To eliminate the adsorbed moisture and volatile impurities of the sample a series of preliminary experiments was carried out at the same temperature. The experiment was finished, when the evaporation rate m/τ became constant with 1 % deviation.

The vapor pressure in the effusion cell *P*_*k*_ was calculated by equation [17]:

9 $$P_{k} = \frac{m}{KS\tau \alpha }\sqrt {\frac{2\pi RT}{M}}$$

where *τ* is the time of effusion through a hole in the membrane with area *S*; *T*—temperature, *R*—universal gas constant, *M*—molecular weight of the substance, *α*—condensation coefficient.

Investigated nitrophenylfuran aldehydes are molecular crystals that sublime without change in their geometry and molecule weight, which also allowed us to admit α to be equal 1. The results of [18], in which equality α = 1 is established for trans-stilbene, benzoic acid, benzophenone, adamantane and other organic compounds by particular measurements of the vapor pressure by three different (torsion, Knudsen and Langmuir) methods serve as the indirect confirmation of this decision validity.

Clausing coefficient—K, which stands for the membrane’s resistance to molecular flow of vapor for the hole in the membrane, which has a ratio of length (l) to radius (r) from 0 to 1.5 was determined by the empirical Kennard formula K = 1/[1 + 0.5(l/r)] [19]:

The vapor pressure was calculated using correction factor according to the recommendations [20]. The correction factor was determined by dependence of benzoic acid vapor pressure on the diameter of 2 membranes at 333 K, analyzed by us and supplemented by the previous analysis of 7 membranes form work [20]. The direct extrapolation to “zero” area allowed calculating the correction multipliers. For the membrane used in the present work, the factor is equal to 1.14.

### Calorimetric measurements

The combustion enthalpies of the aldehydes were determined by upgraded [21] calorimeter V-08MA with isothermal shell. In the ignition system a transformer with a voltage of 40 V, which burned-out wires, was replaced by a set of capacitors with capacity of 2000 µF. This allowed only incandescing the wire and reducing heat heterogeneity. Some experiments showed that energy of the current passing through the wire was registered between 1.4 and 1.7 J. The energy of electric current is four orders of magnitude lower than the combustion energy of substances. Therefore, it was excluded from the calculations.

To reduce the fluctuations of the temperature of thermostatic control the heaters were mounted in series with additional resistance that would be utilized, upon achieving such notion of reduced fluctuations, thus helping to increase the accuracy of ±0.03°.

Irremovable gate systems have been replaced with removable needle closures in a batch calorimeter to ensure the reliability of gas analysis.

The energy equivalent of the calorimetric system W was estimated by combustion of the reference benzoic acid grade K-1 (the major component content—99.995 % mol., heat of combustion, taking into account the Jessup factor—26,434.4 J g^−1^) in a series of 13 experiments. The value W was 15,277.1 ± 8.8 J V^−1^. Experimental error was determined with the Student’s confidence coefficient of 95 %.

Before combustion beginning the crystalline aldehydes were grinded in chalcedony mortar, screened, placed in terylene ampoules and ignited in the quartz cup. A cotton thread tied to platinum wire (d = 0.1 mm) was used for the ampoules ignition. The initial pressure of the oxygen, which was previously purified from the combustible impurities, carbon dioxide and water, was equal to 3.04 × 10^6^ Pa. The initial temperature of the main period in all experiments was 298.15 K, the duration of the main period—20 min. The quantitative analysis of the combustion products for the presence of carbon oxide by the Rossini method [22] with the accuracy of ±2 × 10^−4^ g and also nitric acid content by titration of the liquid phase in a bomb with a 0.1 M solution of NaOH was carried out after every experiment. The quantity of the carbon dioxide, which was formed from the combustion of 1 gram of terylene and the cotton thread, consisted of 2.2872 and 1.6284 g respectively [23]. The anticipated carbon monoxide to be formed during the combustion of products by using detector tubes within ±5 × 10^−6^ g, was not encountered. The soot mass was determined by weighting the quartz cup before and after combustion with the accuracy of ±5 × 10^−6^ g. The reliability of gas analyses was controlled by benzoic acid combustion.

## Conclusions

In the present work the temperature dependence of vapor pressure for all three isomers in the crystalline state and for compound I in a liquid state was determined. The temperature dependences of vapor pressure for compounds II and III in the liquid state are absent because their melting temperatures are (428.6 ± 1) K and (479.8 ± 0.8) K respectively. They are higher than the upper temperature limit of experimental installation (480 K). The values of correlation coefficients ρ (0.9911–0.9988) and the absolute uncertainties of determination of the sublimation enthalpy (2.4–5.7) kJ/mol (Table 2) indicate proper accuracy of the effusional measurements. The absolute values of uncertainties of the combustion enthalpies (6.7–8.0) kJ mol^−1^ confirm acceptable accuracy of measurements for compounds with such complex structure.

The results of this work initiate the research of thermodynamic properties of compounds with arylfurylic fragment. Standard molar enthalpies of formation and sublimation can already be used as a criterion for the possibility to use certain programs or additive schemes for the compounds with arylfurylic fragment.
